# PIP_2_ Mediated Inhibition of TREK Potassium Currents by Bradykinin in Mouse Sympathetic Neurons

**DOI:** 10.3390/ijms21020389

**Published:** 2020-01-08

**Authors:** Paula Rivas-Ramírez, Antonio Reboreda, Lola Rueda-Ruzafa, Salvador Herrera-Pérez, J. Antonio Lamas

**Affiliations:** Laboratory of Neuroscience, Biomedical Research Centre (CINBIO), University of Vigo, 36310 Vigo, Spain; privas84@hotmail.com (P.R.-R.); areboreda@gmail.com (A.R.); lolarrzg@gmail.com (L.R.-R.); ssalva4@me.com (S.H.-P.)

**Keywords:** bradykinin, perforated patch, PIP_2_, riluzole, sympathetic neurons, TREK currents

## Abstract

Bradykinin (BK), a hormone inducing pain and inflammation, is known to inhibit potassium M-currents (I_M_) and to increase the excitability of the superior cervical ganglion (SCG) neurons by activating the Ca^2+^-calmodulin pathway. M-current is also reduced by muscarinic agonists through the depletion of membrane phosphatidylinositol 4,5-biphosphate (PIP_2_). Similarly, the activation of muscarinic receptors inhibits the current through two-pore domain potassium channels (K2P) of the “Tandem of pore-domains in a Weakly Inward rectifying K^+^ channel (TWIK)-related channels” (TREK) subfamily by reducing PIP_2_ in mouse SCG neurons (mSCG). The aim of this work was to test and characterize the modulation of TREK channels by bradykinin. We used the perforated-patch technique to investigate riluzole (RIL) activated currents in voltage- and current-clamp experiments. RIL is a drug used in the palliative treatment of amyotrophic lateral sclerosis and, in addition to blocking voltage-dependent sodium channels, it also selectively activates the K2P channels of the TREK subfamily. A cell-attached patch-clamp was also used to investigate TREK-2 single channel currents. We report here that BK reduces spike frequency adaptation (SFA), inhibits the riluzole-activated current (I_RIL_), which flows mainly through TREK-2 channels, by about 45%, and reduces the open probability of identified single TREK-2 channels in cultured mSCG cells. The effect of BK on I_RIL_ was precluded by the bradykinin receptor (B_2_R) antagonist HOE-140 (d-Arg-[Hyp^3^, Thi^5^, d-Tic^7^, Oic^8^]BK) but also by diC_8_PIP_2_ which prevents PIP_2_ depletion when phospholipase C (PLC) is activated. On the contrary, antagonizing inositol triphosphate receptors (IP_3_R) using 2-aminoethoxydiphenylborane (2-APB) or inhibiting protein kinase C (PKC) with bisindolylmaleimide did not affect the inhibition of I_RIL_ by BK. In conclusion, bradykinin inhibits TREK-2 channels through the activation of B_2_Rs resulting in PIP_2_ depletion, much like we have demonstrated for muscarinic agonists. This mechanism implies that TREK channels must be relevant for the capture of information about pain and visceral inflammation.

## 1. Introduction

Bradykinin (BK) is released during tissue damage, it is one of the main mediators of inflammation and is able to activate and sensitize the nociceptor neurons mainly through the activation of B_2_ receptors (B_2_Rs) [1,2,3]. B_2_Rs are constitutively expressed in most tissues while B_1_ receptors (B_1_Rs) are induced by the inflammatory process [2,3,4,5,6,7,8].

It seems coherent that the sensation of pain produced by BK must be mediated by its action on sensory neurons (nociceptors), whether somatic [2,3] or visceral [9,10]. However, the presence of bradykinin receptors (BRs) in motor neurons of the sympathetic superior cervical ganglion (SCG) was described a long time ago. When studying the effect of BK on the movement of the nictitating membrane of cats, BK was shown to be a potent stimulator of the SCG [11]. Consistently, BK increases the excitability (membrane depolarization at rest and reduction of the spike frequency adaptation (SFA)) of rat SCG (rSCG) neurons [4,12,13,14]. Depolarization by activation of B_2_Rs has also been reported in the entire mouse ganglion [13,15,16]; however, changes in excitability have not been investigated in isolated cultured mouse SCG (mSCG) neurons. The importance of BR expression on SCG neurons is not completely understood. Studies have suggested that depolarization of presynaptic sympathetic-neuron terminals by BK induces the release of prostanoids which acting on nociceptor terminals may induce hyperalgesia. Consistently sympathectomy strongly reduces chloroform-induced hyperalgesia and its exacerbation by BK [4,17].

BK-induced excitability has been ascribed to the inhibition of the potassium M-current and such inhibition was reported to be due to B_2_R and Gαq/11 protein activation in rat and Gα11 in mouse SCG neurons [5,12,18]. The participation of phospholipase C (PLC), inositol triphosphate (IP_3_), Ca^2+^ released from IP_3_ stores and activation of Ca^2+^-Calmodulin in this process was also reported in rSCG [14,19,20]. Traditionally, the regulation of the potassium M-current by muscarinic agonists has been considered the main physiological pathway to modulate the excitability of SCG neurons [21,22,23,24]. However, researchers have also reported that the same agonists do modulate background two-pore domain potassium (K2P) channels of the “Tandem of pore-domains in a Weakly Inward rectifying K^+^ channel (TWIK)-related channels” (TREK) subfamily in these cells [25]. The two most expressed K2P channels in mSCG neurons are “TWIK-related spinal cord potassium channel” (TRESK) and TREK-2 [26,27] and it was reasonable to hypothesize that if the activation of muscarinic receptors inhibits M and TREK currents (I_TREK_) in mSCG, the activation of their bradykinin counterparts could do the same. Indeed, both types of channels, but also both types of receptors, have been thoroughly studied due to their important role on the setting of the resting membrane potential (RMP) and on the modulation of membrane excitability in many cell types. 

The study of TREK currents in native neurons is challenging because the activity of these channels is reduced at room temperature and atmospheric pressure [28,29,30]. We take advantage of the neuroprotective agent riluzole, which activates the three members of the TREK subfamily (TREK-1, TREK-2 and TRAAK (TWIK-related arachidonic acid-stimulated potassium channel)) [31,32,33,34]. Riluzole does not activate other K2P channels and it was demonstrated that the outward current evoked by riluzole in mSCG neurons is mainly driven through TREK-2 channels [25,26,35]. TREK-1 and TREK-2, but not TRAAK, are well known to be modulated through G-protein coupled receptors and PLC activation [36,37]. In fact, it has been proposed that basal G protein activity may have a constant down regulating effect on these channels [38,39].

We have reported before that, in mSCG neurons, the muscarinic inhibition of TREK-2 currents following the PLC pathway requires a reduction of phosphatidylinositol 4,5-biphosphate (PIP_2_) levels [25]. In the current study we demonstrate that, much like it was reported in rSCG neurons, BK depolarizes and increases the excitability of mSCG neurons. A good amount of evidence is given to show that BK inhibits a riluzole activated TREK-like current by reducing the membrane PIP_2_ levels, via the same pathway than muscarinic agonists in the same preparation. The presence of this newly-described current could explain, at least in part, the effects of BK in SCG neurons. The main achievement of this study was to discover a new target for the control of sympathetic excitability by bradykinin. TREK channels are known to regulate the resting membrane potential in a good number of cellular types [34].

## 2. Results

Bradykinin has been shown to strongly modulate neuronal excitability by reducing M-currents in several preparations [40] including rat SCG neurons but, to our knowledge, only one study [41] has indirectly related bradykinin with TREK channels. Voltage-clamp whole-cell experiments were performed at room temperature (22–24 °C) and holding the neurons at −30 mV. A blocking “cocktail” containing tetraethylammonium (TEA, 15 mM), tetrodotoxin (TTX, 0.5 µM), and CsCl (1 mM) was used to block M-currents, voltage-dependent Na^+^ currents, and the hyperpolarization activated cationic h-current (I_h_), respectively [42,43]. This blocking cocktail reduced the basal outward current found at −30 mV (I_-30_) by about 40% [25] and does not affect the riluzole-activated outward current as previously demonstrated [26].

### 2.1. Bradykinin Increases the Excitability of mSCG Neurons

The resting membrane potential of mSCG neurons recorded in culture was −62.6 ± 1.3 mV (*n* = 33), and in order to reduce variability, we manually clamped neurons at −60 mV before applying any treatment or protocol. For the same reason, only neurons firing less than 10 action potentials in response to maximal 1 s depolarizing pulses (adapting cells), were analyzed in this study [44]. 

**Figure 1:**
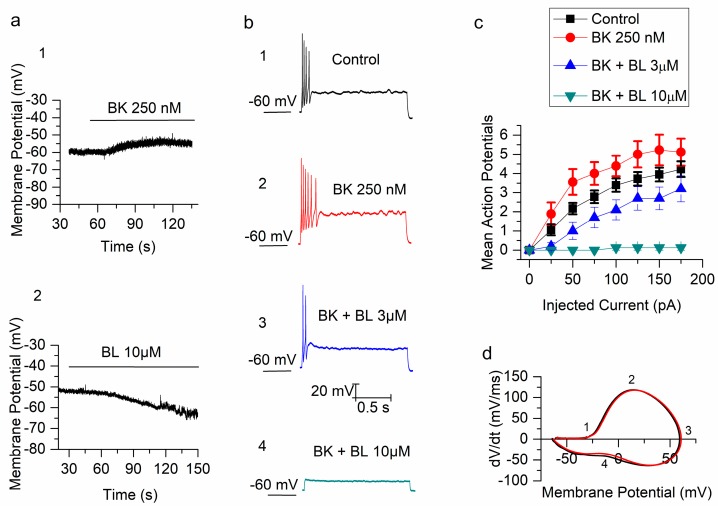
Bradykinin (BK) increases excitability in mouse superior cervical ganglion (mSCG) neurons. (**a**) The bradykinin depolarized (**1**) and BL1249 (5,6,7,8-tetrahydro-naphthalen-1-yl) -[2-(1*H*-tetrazol-5-yl)-phenyl]-amine) hyperpolarized (**2**) membrane potential of mSCG neurons. (**b**) The firing pattern evoked by a 125 pA current injection, in a mSCG neuron, in control (**1**) and after the application of BK (**2**), BK + BL − 3 µM (**3**) and BK + BL − 10 µM (**4**). (**c**) The number of action potentials (mean ± SEM) evoked by depolarizing step currents from 25 to 175 pA in 25 pA increments in four conditions: control (squares), BK 250 nM (circles), BK + BL 3 µM (triangles) and BK + BL 10 µM (inverted triangles). (**d**) Phase plot for two action potentials before and after BK 250 nM.

In those conditions, bath application of bradykinin 250 nM depolarized mSCG neurons by 6.3 ± 0.7 mV (*n* = 18, *p* < 0.001, Figure 1(a1)) and in three of them, BK induced cell firing (not shown). As expected, application of the recently discovered activator of TREK channels BL1249 (BL, (5,6,7,8-tetrahydro-naphthalen-1-yl)-[2-(1*H*-tetrazol-5-yl)-phenyl]-amine) [45,46,47] had an opposite effect on the resting membrane potential hyperpolarizing mSCG neurons by −5.7 ± 0.9 mV (*n* = 7, *p* < 0.01) and −17.6 ± 1.5 mV (*n* = 8, *p* < 0.001, Figure 1(a2)), when applied at 3 µM and 10 µM respectively. When BL (3 and 10 µM) was applied in the presence of bradykinin 250 nM, it produced a similar and significant hyperpolarization: −8.4 ± 0.7 mV (*n* = 10, *p* < 0.001) and −18.7 ± 1.5 mV (*n* = 8, *p* < 0.001), respectively (not shown). Interestingly, when applied in the presence of BL (3 and 10 µM), the depolarization produced by BK 250 nM was not statistically different between both groups (6.5 ± 1.6 and 4.5 ± 0.8 mV, *p* > 0.05, *n* = 7 and 8 respectively) and they were also not different from the control (only BK, *p* > 0.05). BL1249 has been shown to activate TREK-1 and TREK-2 channels but no other K2P channels [45].

mSCG neurons are well known for their strong SFA in response to injections of depolarizing current (Figure 1(b1)) in standard solutions. Bradykinin (250 nM) provoked a small but significant increment (*p* < 0.05, *n* = 18) of the number of action potentials (Figure 1(b2)) in response to depolarizing current injections from 25 to 175 pA (Figure 1c). In the presence of BK, BL1249 reduced the firing at both 3 and 10 µM (*n* = 10 and 8 respectively; *p* < 0.05; Figure 1(b3,b4),c). The effect of BL 10 µM was so dramatic that neurons were unable to respond at all (Figure 1(b4),c). Also when BL 10 µM was applied first, mSCG neurons stopped firing at any current injection (*n* = 8, *p* < 0.05) and subsequent application of BK (in the presence of BL) did not increase the excitability (not shown). The effect of BL 3 µM on the firing was not significant but interestingly it precluded the increase of firing normally produced by BK 250 nM (*n* = 5; *p* > 0.05).

In order to investigate the effect of BK on the action potentials of mSCG neurons, we constructed phase plots calculating the derivative of voltage with respect to time and plotting it against membrane voltage. Figure 1d shows that the action potential threshold (1), maximal up-stroke velocity (2), maximal positive voltage reached (3), and hump decrease in velocity (4) were indistinguishable before and after application of BK 250 nM. In fact, the amplitude of the action potential (121.75 ± 3.01 vs. 120.72 ± 2.78 mV, *n* = 17, *p* = 0.1), the half-amplitude duration (2.37 ± 0.15 vs. 2.24 ± 0.12 ms, *n* = 17, *p* = 0.16), the threshold (−35.07 ± 1.16 vs. −35.14 ± 1.12 mV, *n* = 17, *p* = 0.88) and the latency to the first action potential in response to depolarizing current injections at 50 pA (28.53 ± 8.94 vs. 20.61 ± 1.98 ms, *n* = 17, *p* = 0.39) were all not statistically different.

### 2.2. Bradykinin Inhibits Whole-Cell and Single-Channel TREK-2 Currents

**Figure 2:**
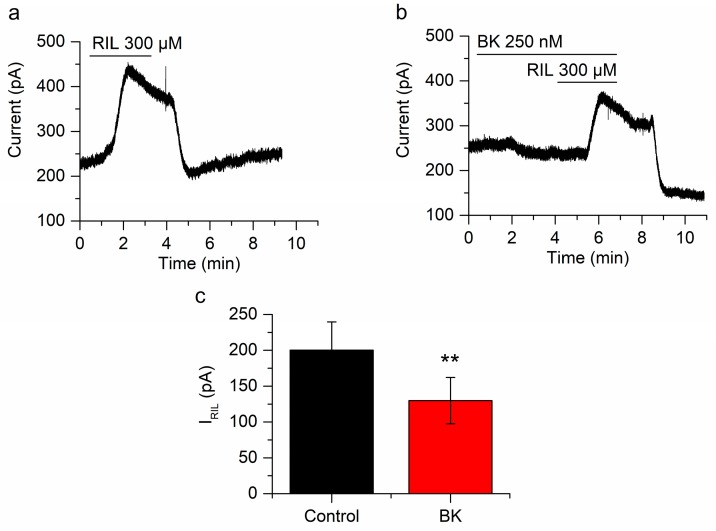
Bradykinin inhibits I_RIL_. (**a**) Outward current induced by the application of riluzole (RIL) (300 µM) in mSCG neurons fixed at −30 mV (I_RIL_). (**b**) Both, the current at −30 mV and I_RIL_ are reduced in the presence of BK (250 nM). (**c**) The difference between I_RIL_ control (200.2 ± 39.3 pA, *n* = 7, black bar) and I_RIL_ in the presence of bradykinin (129.8 ± 32.24 pA, *n* = 7, red bar) was clear and significant (*p* < 0.01). Recordings in (**a**,**b**) belong to the same neuron. ** *p* < 0.01.

To obtain the control I_RIL_ (200.2 ± 39.3 pA; *n* = 7; Figure 2a) we applied riluzole (300 µM for at least 3 min) to neurons clamped at −30 mV in the whole-cell perforated-patch mode and in the presence of the blocking cocktail. After washing out riluzole we applied BK (250 nM) during 4 min before the second application of riluzole, which evoked an I_RIL_ significantly smaller than I_RIL_ control (129.8 ± 32.2 pA, *n* = 7; Figure 2b), showing a significant reduction of 43.2 ± 6.1% (*n* = 7; *p* < 0.01; Figure 2c). Additionally, BK reduced the I_-30_ in 83.8 ± 24.1 pA (*n* = 7; Figure 2b). We have demonstrated before that repetitive application of riluzole does not desensitize I_RIL_ [25].

**Figure 3:**
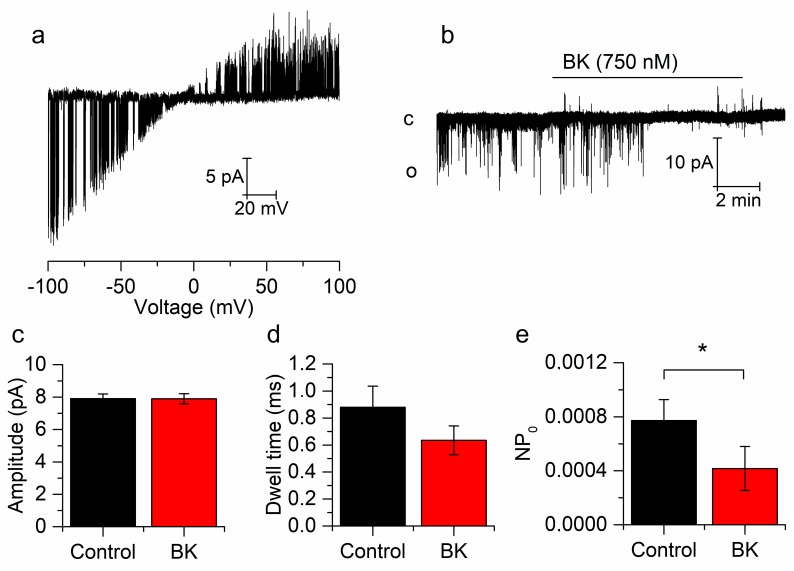
Bradykinin reduces the open probability of single TREK-2 channels. (**a**) Representative cell-attached, TREK-2 single-channel behavior in response to voltage ramps from −100 to +100 pA in mSCG neurons. (**b**) Effect of BK on a single TREK-2 channel voltage-clamped at −60 mV. (**c**–**e**) BK reduces the open probability (**e**) without affecting dwell time (**d**) or current amplitude (**c**). Recordings belong to the same patch. * *p* < 0.05.

Single TREK-2 channels were identified by using voltage ramps from −100 to +100 mV in SCG neurons. Cell-attached recordings showed a high conductance at negative potentials, which was clearly reduced at positive voltages, which is characteristic of TREK-2 channels (Figure 3a). Holding the patch (cell-attached) at −60 mV we applied BK (750 nM) in order to investigate whether BK can affect TREK-2 channels using an indirect pathway through second messengers (Figure 3b). The amplitude, open dwell time (duration of channel openings) and open probability (NP_o_) were measured from one minute recordings. These recordings were taken before BK application (control) and at least 3 min after BK application (BK), when the effect of the drug was stabilized. Figure 3e shows that BK induced a reduction in the NP_o_ (from 7,72E-4 ± 1,56E-4 to 4,17E-4 ± 1,63E-4, *p* < 0.05, *n* = 11) without affecting the current amplitude (from 7.9 ± 0.3 pA to 7.9 ± 0.3 pA, *p* = 0.979, *n* = 11, Figure 3c) nor the open dwell time (from 0.88 ± 0.14 ms to 0.71 ± 0.12 ms, *p* = 0.290, *n* = 11, Figure 3d), indicating that BK reduced I_RIL_ by reducing the open probability of the TREK-2 channels. These experiments strongly indicated that BK can inhibit TREK-2 channels and hence I_RIL_ through the activation of a second messenger cascade.

### 2.3. The Inhibition of I_RIL_ by BK Is Mediated through B_2_ Receptors

The binding of BK to specific receptors, mainly B_1_R and B_2_R, provokes a signalling cascade starting with G-protein activation. In rat sympathetic neurons, G_q/11_ is the main G-protein implicated in BK modulation [12]. In turn, G_q/11_ activates the enzyme PLC which hydrolyzes PIP_2_ to give IP_3_ and diacylglycerol (DAG). IP_3_ binds to the IP_3_ endoplasmic reticulum receptors releasing Ca^2+^ and therefore increasing its intracellular concentration [48]. Finally, the Ca^2+^ increase, together with DAG, activates the PKC, which can phosphorylate other proteins [49]. All intermediate products of this cascade have been shown to directly modulate ion channels in different preparations.

**Figure 4:**
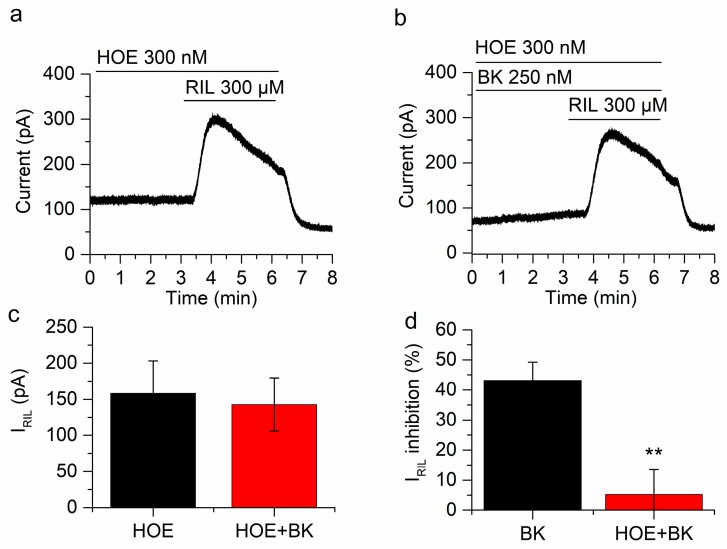
BK inhibition of I_RIL_ is mediated by B_2_R. (**a**) I_RIL_ was not affected in the presence of HOE-140 (d-Arg-[Hyp3, Thi5, d-Tic7, Oic8]BK) in mSCG neurons held at −30 mV. (**b**) In the presence of HOE-140 the inhibition of I_RIL_ by BK was significantly reduced. (**c**) There is no difference between I_RIL_ control (158.3 ± 44.9 pA, black bar) and I_RIL_ in the presence of BK and HOE-140 (142.7 ± 36.8 pA). (**d**) The inhibition by BK in the presence of HOE-104 was 5.2 ± 8.3% (red bar), which is significantly different from the inhibition in the absence of HOE-140 (43.2 ± 6.1%, black bar, *p* < 0.01). Recordings in (**a**,**b**) belong to the same neuron. ** *p* < 0.01.

**Figure 5:**
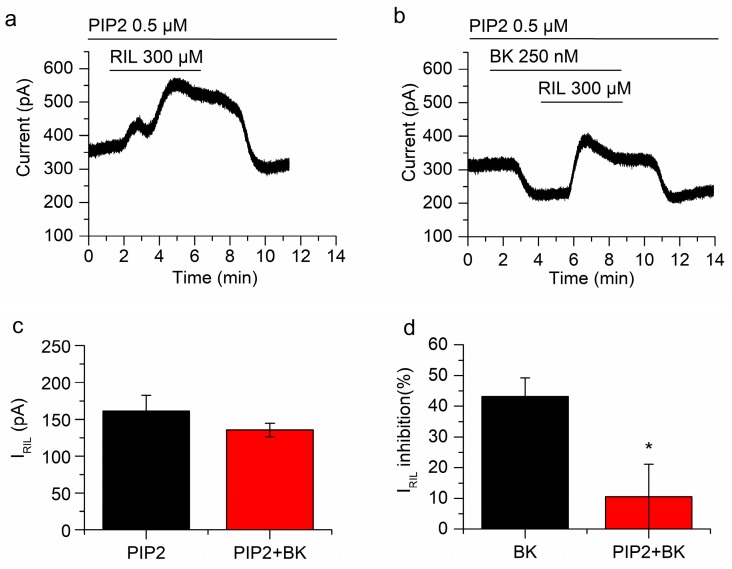
Keeping phosphatidylinositol 4,5-biphosphate (PIP_2_) concentration precludes the inhibition of TREK-2 by BK. (**a**) Riluzole-activated current after incubation of neurons with diC_8_PIP_2_ for 1 h. (**b**) In the presence of a PIP_2_ excess, BK clearly reduced I_-30_ but the inhibition of I_RIL_ by BK was abolished. (**c**) There was no difference between I_RIL_ (black bar; *n* = 5) and I_RIL_ in the presence of BK (red bar; *n* = 5) when cells were pre-incubated with diC_8_PIP_2_. (**d**) The inhibition of I_RIL_ by BK in the presence of diC_8_PIP_2_ (red bar; *n* = 5) is significantly different (*p* < 0.05) from the inhibition by BK alone (black bar; *n* = 7). Recordings in (**a**,**b**) belong to the same neuron. * *p* < 0.05.

**Figure 6:**
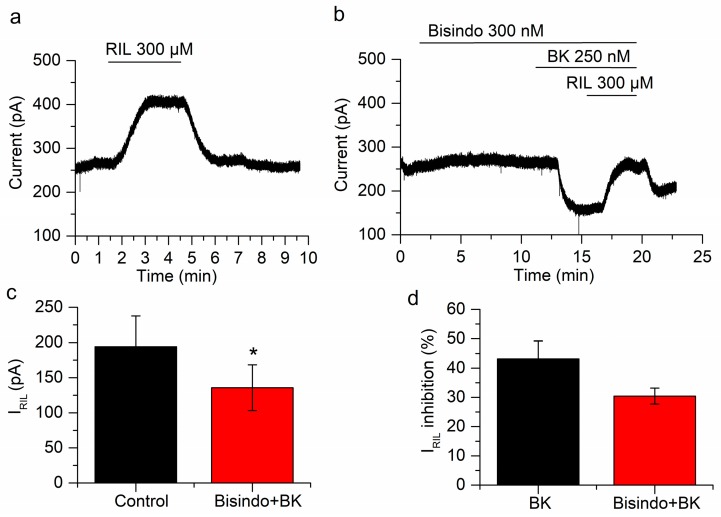
Protein kinase C (PKC) does not affect the inhibition of I_RIL_ by BK. (**a**) Current activated by riluzole in control conditions. (**b**) In the presence of the PKC blocker bisindolylmaleimide both I_-30_ and I_RIL_ are inhibited by BK. (**c**) The difference between I_RIL_ (black bar) and I_RIL_ in the presence of bisindolylmaleimide and BK (red bar) is statistically significant (*p* < 0.05; *n* = 4). (**d**) The inhibition of I_RIL_ by BK in presence of bisindolylmaleimide (red bar) is similar to the inhibition by BK alone (black bar; *p* = 0.17). Recordings in (**a**,**b**) belong to the same neuron. * *p* < 0.05.

**Figure 7:**
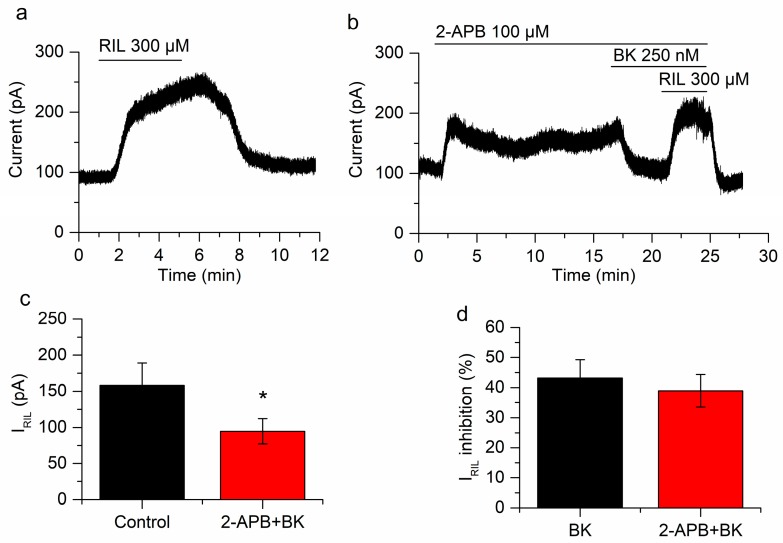
Ca^2+^ release does not play a role in the inhibition of I_RIL_ by BK. (**a**) I_RIL_ in control conditions. (**b**) Application of 2-aminoethoxydiphenylborane (2-APB) provokes an outward current at −30 mV and the inhibition of I_RIL_ by BK is not affected in the presence of this drug. (**c**) The difference between I_RIL_ control (black bar) and I_RIL_ in presence of BK and 2-APB (red bar) is significant (*p* = 0.029; *n* = 5). (**d**) The percentages of BK inhibition in absence (black bar) and presence of 2-APB (red bar) are similar (*p* = 0.79). Recordings in (**a**,**b**) belong to the same neuron. * *p* < 0.05.

To investigate the participation of BK receptors in the inhibition of I_RIL_, we used the selective B_2_R antagonist HOE-140 (d-Arg-[Hyp^3^, Thi^5^, d-Tic^7^, Oic^8^]BK). In a first step we applied riluzole (300 µM for 3 min in the presence of cocktail + HOE) to obtain I_RIL_ in control conditions (158.3 ± 44.9 pA; *n* = 4; Figure 4a). Interestingly, the application of HOE (300 nM) to the bath solution slightly increased the steady-state current at −30 mV in 12.2 ± 1.2 pA (*n* = 4; not shown). Bradykinin (250 nM) was then applied in the presence of cocktail + HOE, it is relevant that in the presence of the B_2_ receptor antagonist, BK did not reduce the outward current at −30 mV (See panels b in Figure 5, Figure 6 and Figure 7). Finally, a second application of riluzole (300 µM, 3 min) in cocktail + HOE + BK (Figure 4b), induced an outward I_RIL_ showing no significant difference with the I_RIL_ control (142.7 ± 36.8 pA; *n* = 4; *p* = 0.2804; Figure 4c). The percentage of inhibition of I_RIL_ by BK in presence of HOE was insignificant (5.2 ± 8.3%; Figure 4d), clearly indicating that the inhibition of I_RIL_ by BK can be entirely explained by the activation of B_2_ receptors.

### 2.4. Manipulating PIP_2_ Concentration Affects the Inhibition of I_RIL_ by Bradykinin

We have recently reported that the inhibition of TREK-2 channels by muscarinic agonists in mSCG neurons greatly depends on PIP_2_ depletion [25]. As B_2_ receptors also activate G_q/11_ and PLC, the reduction of PIP_2_ seemed a good candidate to explain the inhibition of I_RIL_ by BK. To investigate this issue, we incubated mSCG neurons with diC_8_PIP_2_ (0.5 µM, saturating concentration) and a histone carrier, for 1 h, before starting current recordings. As expected, in this situation, the application of BK did not affect I_RIL_ significantly (From 161.3 ± 21.4 pA to 135.5 ± 9.3 pA, *p* = 0.20, *n* = 5; Figure 5a,b). Consistently, the percentage of current reduction (10.5 ± 10.6%) was irrelevant when compared with the control (43.2 ± 6.1%; without diC_8_PIP_2_; Figure 5c,d). Nevertheless, BK inhibited I_-30_ significantly (−113.4 ± 22 pA; *p* = 0.0067; *n* = 5; Figure 5b) in presence of diC_8_PIP_2_ indicating distinct pathways involved in the inhibition of I_RIL_ and I_-30_ (experiments carried out in cocktail). Altogether these results demonstrate that the inhibition of the riluzole activated current by bradykinin is mediated by the reduction of PIP_2_ but the inhibition of I_-30_ is not.

### 2.5. Neither Protein Kinase C nor Ca^2+^ Are Involved in the Inhibition of I_RIL_ by Bradykinin

In the PLC pathway, concomitant with the reduction of PIP_2_ there is an increase of IP_3_ and DAG. DAG and the Ca^2+^ released by the binding of IP_3_ to its receptor finally activate PKC. Indeed, research has shown that the inhibition of the M-current by BK depends on the increase of Ca^2+^ produced in this way [19,50]. We investigated the role of these last messengers, on the inhibition of TREK-2 currents by BK, by inhibiting PKC and antagonizing IP_3_ receptors.

In order to inhibit PKC we applied bisindolylmaleimide (300 nM) for 10 min. In these conditions, bradykinin inhibited I_RIL_ by 30.4 ± 2.7%, from 194.1 ± 43.5 to 135.7 ± 32.6 pA (*p* = 0.023; *n* = 4; Figure 6a–c). This inhibition is not different (*p* = 0.17) from that obtained in the absence of bisindolylmaleimide (43.2 ± 6.1%; Figure 6d). The application of bisindolylmaleimide had no effect on I_-30_ (7.4 ± 4.3 pA; *n* = 4; *p* = 0.18; Figure 6b) but BK application reduced significantly this current (−81 ± 22.1 pA; *p* = 0.035; *n* = 4; Figure 6b). This experiment indicated that PKC is not involved in the inhibition of I_RIL_ or I_-30_ by BK.

To test the other limb of the PLC pathway we antagonized the IP_3_R using 2-APB (100 µM) and hence hampered the increase in Ca^2+^ that is normally produced by the activation of BK receptors. Bradykinin still reduced I_RIL_ when the increase in Ca^2+^ was prevented (from 158.4 ± 30.7 pA to 94.6 ± 17.5 pA; *p* = 0.029; *n* = 5; Figure 7). The inhibition (39 ± 5.4%; Figure 7d) was similar to that found in the absence of 2-APB (43.2 ± 6.1%; *p* = 0.79; Figure 7d). We previously performed control experiments comparing riluzole activation in the presence and absence of 2-APB and differences were not found [26]. Interestingly, the application of 2-APB produced a fast increase of I_-30_ (71.4 ± 19.3 pA; *p* = 0.020; Figure 7b), this was a transient effect that slowly decreased in −29.9 ± 9.7 pA (*p* = 0.037). This was in agreement with 2-APB acting as a TREK-2 activator [51]. The application of BK (250 nM) still reduced I_-30_ in the presence of 2-APB (−48.3 ± 1.5 pA; *p* = 5.18 × 10^−6^; *n* = 5; Figure 7b), probably reflecting the inhibition of the TREK-2 current, previously activated by 2-APB.

## 3. Discussion

In addition to being a potent vasodilator and a pain and inflammation activator [1], bradykinin depolarizes the resting membrane potential and reduces the spike frequency adaptation in rSCG neurons [12,13]. This modulation has been related to the M-current that is well known to be inhibited by BK [12,50,52]. On the other hand, the potassium channels of the TREK subfamily have a similar role to the M channels in some excitable cells, contributing to the control of the RMP, the firing and therefore to the control of excitability [26,29]. In the present work we demonstrated, for the first time, that bradykinin also modulates TREK currents in mSCG neurons by reducing PIP_2_. The inhibition of these channels could contribute to the increase in excitability produced by BK in sympathetic neurons.

### 3.1. I_-30_ Includes I_TREK_ and I_M_

When the membrane of SCG neurons is depolarized to −30 mV, a characteristic and constant outward current emerges, this current was initially attributed to the opening of voltage-activated, non-inactivating potassium M-channels and it explains why most voltage protocols used to study the M-current start by fixing neurons at −30 mV [53]. Our group has demonstrated the expression of background potassium TREK-2 currents in mSCG neurons but although these are considered essentially voltage-independent, its macroscopic intensity-voltage curve is almost indistinguishable from that of the M-current [25,35]. The best tool we have to isolate the TREK current from the M-current is to selectively activate TREK currents with riluzole in the presence of TEA, a strong blocker of the M-currents but not affecting TREK channels [26]. We have previously demonstrated that the current activated by riluzole is not affected by blockers of other channels like voltage-gated and persistent Na^+^, voltage-gated Ca^2+^, hyperpolarization-activated cation, transient-receptor- potential cation, voltage-gated A and DR type K^+^, M type K^+^, and Ca^2+^-activated K^+^ (Big K^+^ and Small K^+^) channels [26]. As riluzole activates the TREK subfamily exclusively [26,31,32], and, within the TREK subfamily, TREK-2 is largely more expressed than the other TREK subfamily channels in mSCG neurons [27], we can propose that I_RIL_ is mainly transported through TREK-2 channels in these neurons. Assuming that the outward current observed at −30 mV is composed mainly of I_M_ and I_TREK_, when TEA and other blockers not affecting TREK currents are applied, we expect the remaining current to be mainly I_TREK_. Even so, we call this current I_-30_ because we suspect that the cocktail may not be capable of removing the I_M_ completely.

### 3.2. I_RIL_ is Due to the Activation of I_TREK_

As BK inhibits both the M and TREK currents, the question arises of whether I_RIL_ can be contaminated with the M-current. BK applied in the cocktail reduces I_-30_ and inhibits I_RIL_, and both effects disappear when the BK receptors are antagonized, indicating that both depend on BK receptor activation. This suggests that part of the I_-30_ is due to I_TREK_ as most, if not all, I_M_ is blocked by TEA [43]. 

On the contrary, when we keep high levels of PIP_2_, BK is not inhibiting I_RIL_ but still reduces I_-30_, suggesting that BK inhibits I_RIL_ by depleting PIP_2_ but it is able to reduce part of the I_-30_ (probably I_M_) through a different pathway when PIP_2_ reduction is not possible. Taken together all these data indicate that I_RIL_ is essentially a riluzole-activated TREK current and that I_-30_ has two components (I_TREK_ and a residual I_M_ when TEA is present). This hypothesis is strongly supported by the fact that I_M_ has been reported to be inhibited by BK through the activation of Ca^2+^-calmodulin, but not by PIP_2_ depletion (like reported for muscarinic agonists), in SCG neurons [12,52,54,55]. This different mechanism could be due to the fact that muscarinic receptors are far away from IP_3_R and hence Ca^2+^ release from the ER becomes difficult. However, BK receptors are close enough to provoke Ca^2+^ release [52,56]. Keeping a high level of PIP_2_ does not impede IP_3_ production and hence Ca^2+^ release, when BK stimulates its receptors [19]. 

Moreover, as we reported before, when using the PI3/PI4-Kinase-inhibitor wortmannin to block the replenishment of PIP_2_ and to maintain PIP_2_ concentration very low it can be demonstrated that I_RIL_, and also I_-30_, need high concentrations of PIP_2_ to be activated. This is consistent with both M and TREK channels being inhibited by PIP_2_ depletion in SCG neurons [25,57]. Our results contrast with previous data showing that BK does not increase the concentration of PIP_2_ in rat SCG neurons [14,58,59]. This discrepancy could be related to the use of different species and ages for the extraction of neurons. In fact, it has also been shown that the regulation of the M current by muscarinic agonists is quite different in rat (G_q_) and mouse (G_11_, G_q_ and PTX-sensitive G-proteins) and not less important, the inhibition of the M current by BK is mediated by G_α11_ in mSCG but by G_αq/11_ in rSCG neurons [18].

### 3.3. I_-30_ versus I_TREK_

When the activation of IP_3_ receptors was avoided with 2-APB and hence prevented the inhibition of I_M_, BK still inhibited I_-30_, indicating that besides the M component there exists a second component that can be inhibited through a pathway different from the Ca^2+^-calmodulin one, probably the TREK current activated by 2-APB contributes to this inhibition. Consistently, the reduction of the I_-30_ in these conditions resulted smaller than that obtained in cocktail alone (around 50 pA) as it represents the I_TREK_ only while the inhibition of I_RIL_ by BK was well preserved in those conditions.

### 3.4. Physiological Relevance and Conclusions

The TREK subfamily has been implicated in the transduction of mechanical, chemical, and thermal stimuli [60,61], but also related to pain transduction, as it is well expressed in dorsal root ganglia (DRG) small neurons and in trigeminal ganglia neurons [62,63,64,65]. Recent studies have proposed the TREK subfamily as a possible target for pain treatment [62,66,67] and TREK-1 seems to be involved in morphine analgesia without adverse effects in mice [68]. Also aristolochic acid, used traditionally as painkiller, enhanced TREK-1 and TREK-2 currents [69]. The present work shows that TREK-2 channels are inhibited by BK, an inductor of pain and inflammation, much like as described before for M-current inhibition [12]. The activation of TREK channels by the neuroprotector riluzole hyperpolarizes nociceptive DRG neurons resulting in a decrease of the effects of BK [70]. The TREK subfamily is well expressed in the DRG neurons [28,71,72], the TREK-2 channel being the most expressed, at least in neonatal rats [73]. Additionally, the blood vessel vasodilation produced by bradykinin is strongly attenuated in TREK-1 deleted animals [41]. These and other studies strongly relate BK with TREK channels and pain.

**Figure 8:**
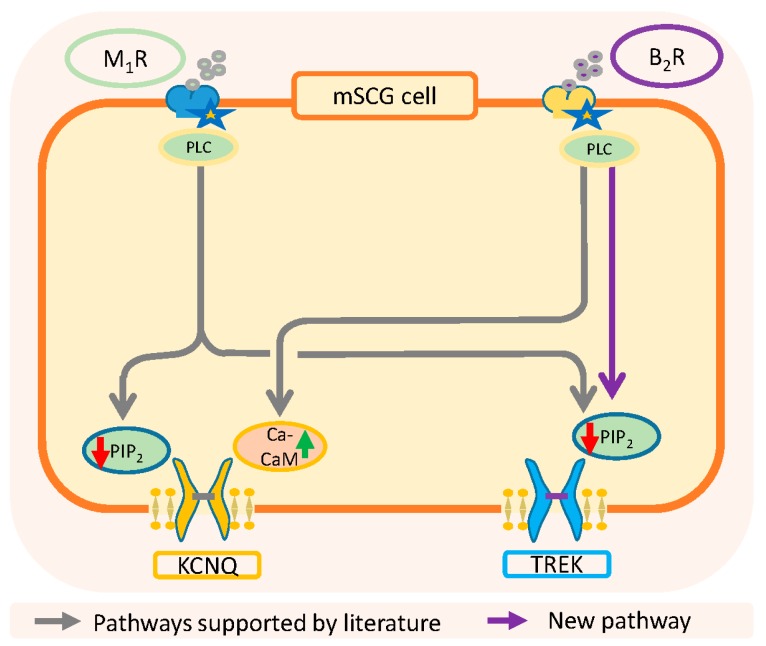
Summary of classical and new pathways involved in the resting membrane potential and excitability of SCG neurons. KCNQ: gen codifying M-type voltage dependent K channels.

As summarized in Figure 8, the activation of muscarinic M_1_-receptors inhibits both I_M_ and I_TREK_ through the depletion of PIP_2_, while the activation of B_2_-receptors uses two different inhibitory pathways, PIP_2_ reduction for I_TREK_ and Ca^2+^-calmodulin for I_M_. Importantly, it has been reported that TREK-1 channels have a group of cationic aminoacids in the C-terminal region that interacts electrostatically with the inner part of the membrane. A reduction in the concentration of PIP2 causes fluctuations in this electrostatic interaction resulting in the dissociation of the c-terminal domain from the membrane and a change in channel activity [74,75]. An important conclusion of this work is that the riluzole activated current and about half of the current at −30 (in the presence of Na^+^, K^+^ and h-current blockers) are carried through TREK channels. Both currents and also individual TREK-2 channels are inhibited by bradykinin through PIP_2_ depletion. These results also suggest that previous interpretations on the effects of BK on the nervous system may need a review.

## 4. Materials and Methods

All protocols performed were approved by the Spanish Research Council and the Animal Welfare Committee of the University of Vigo (Code: 07/2014; Date: 24/10/2016). They accomplished the Spanish (RD 53/2013) and European (2010/63/EU) directives for the protection of animals used for experimental purposes.

### 4.1. Cell Culture

Swiss-CD1 mice of both sexes and 20 to 60-days-old were terminally anaesthetized with CO_2_ before decapitation. SCG ganglia were then quickly dissected and placed in L-15 culture medium to clean and disaggregate, first enzymatically and then mechanically. Then, neurons were placed in laminin-coated 35 mm Petri dishes and kept in culture (37 °C, 5% CO_2_) for 12 to 24 h before recording. Detailed dissection and culture procedures have been reported before [22,76].

### 4.2. Perforated-Patch Recording

Electrophysiological data were recorded using an Axopatch 200B amplifier (Molecular Devices, Union City, CA, USA) controlled through a DIGIDATA 1440A digitizer (Molecular Devices). The software pClamp 10.0 (Molecular Devices) was used to design the protocols. The standard bath solution was composed of (mM): 140 NaCl, 3 KCl, 1 MgCl_2_, 2 CaCl_2_, 10 D-glucose, 10 HEPES (4-(2-hydroxyethyl)-1-piperazineethanesulfonic acid); pH 7.2 adjusted with Tris (Tris(hydroxymethyl)-aminomethane). The intracellular solution contained (mM): 90 K-acetate, 20 KCl, 3 MgCl_2_, 1 CaCl_2_, 3 EGTA (ethylene glycol-bis(β-aminoethyl ether)-*N*,*N*,*N*′,*N*′-tetraacetic acid), 40 HEPES, and ~20 NaOH to achieve a pH of 7.2. Amphotericin-B (75 µg/mL) was freshly prepared every day and added to the intracellular solution to obtain the perforated patch configuration. Borosilicate glass was used to produce 4–5 MΩ electrode resistances. After 15–20 min, since the gigaseal was attained, access resistance reached values below 20 MΩ and recording started. Cells with higher access resistance were discarded. Voltage-clamp recordings were sampled at 2 KHz and low-pass filtered at 1 KHz while current-clamp (bridge-mode like) signals were sampled at 10 KHz filtering at 5 KHz. The data obtained (mean ± SEM) were analyzed and plotted using pClamp 10.0 and Origin 9.0 (OriginLab Corporation, Northampton, MA, USA) and the statistical significance was assumed when paired Student’s *t*-test gave *p*-values less than 0.05.

### 4.3. Single-Channel Recording

Single-channel recordings were performed in the cell-attached configuration. Bath and pipette solutions were identical and contained (mM): 150 KCl, 1 MgCl_2_, 5 EGTA, and 10 HEPES, pH 7.2 was achieved with KOH. The electrode resistance for single-channel experiments ranged from 10 to 12 MΩ. Data were sampled at 20 KHz and low-pass filtered at 2 KHz using the amplifier built-in filter. Single-channel openings faster than 50 µs were discarded. The threshold detection for single-channel openings was set at 50% of the amplitude. Single-channel parameters: amplitude, open dwell time and NP_o_ were measured using pClamp 10.0 software. The NP_o_ was calculated according to the following equation: NP_o_ = t_o_/T, where N is the number of channels, P_o_ is the open state probability, t_o_ was the total time that the channel was found in the open state, and T is the total observation time. The data obtained (mean ± SEM) were analyzed and plotted using Clampfit 10 and Origin 9.0 applying Paired Student’s *t*-test and considering significant *p*-values < 0.05.

### 4.4. Drugs

All drugs were applied directly to the bath solution (10 mL/min) during the protocol, except for diC_8_PIP_2_ with a histone carrier, which was previously added to the culture dishes.

TTX and HOE-140 were purchased from Tocris Bioscience (Bristol, UK), diC_8_PIP_2_ and the histone carrier were purchased from Echelon Biosciences (Salt Lake City, UT, USA), and all the other chemicals were obtained from Sigma-Aldrich (Madrid, Spain).

## Abbreviations

2-APB	2-aminoethoxydiphenylborane
BK	bradykinin
BL	BL1249
BRs	bradykinin receptors
B_1_R	bradykinin receptor 1
B_2_R	bradykinin receptor 2
DAG	diacylglycerol
DRG	dorsal root ganglia
HOE	HOE-140. d-Arg-[Hyp^3^, Thi^5^, d-Tic^7^, Oic^8^]BK
I_h_	hyperpolarization activated cationic current
I_M_	potassium M-current
IP_3_	inositol triphosphate
IP_3_R	inositol triphosphate receptor
I_RIL_	riluzole-activated current
I_TREK_	potassium current through TREK channels
I_-30_	basal outward current at -30 mV
K2P	two pore domain potassium channels
mSCG	mouse superior cervical ganglion
NPo	open probability
PIP_2_	phosphatidylinositol 4,5-bisphosphate
PKC	protein kinase C
PLC	phospholipase C
RMP	resting membrane potential
rSCG	Rat superior cervical ganglion
SCG	superior cervical ganglion
SFA	spike frequency adaptation
TEA TRAAK	Tetraethylammonium TWIK-related arachidonic acid-stimulated potassium channel
TREK TRESK TWIK	TWIK-related potassium channel TWIK-related spinal cord potassium channel Tandem of pore-domains in a Weakly Inward rectifying K^+^ channel
TTX	tetrodotoxin
